# Beyond Chatbots: Moving Toward Multistep Modular AI Agents in Medical Education

**DOI:** 10.2196/76661

**Published:** 2025-10-02

**Authors:** Minyang Chow, Olivia Ng

**Affiliations:** 1 Group Clinical Education, National Healthcare Group, 1 Mandalay Rd, Singapore, 308205, Singapore, 65 6496-6000; 2 Lee Kong Chian School of Medicine, Nanyang Technological University, Singapore, Singapore

**Keywords:** artificial intelligence, agents, pedagogy, large language models, instructional design

## Abstract

The integration of large language models into medical education has significantly increased, providing valuable assistance in single-turn, isolated educational tasks. However, their utility remains limited in complex, iterative instructional workflows characteristic of clinical education. Single-prompt AI chatbots lack the necessary contextual awareness and iterative capability required for nuanced educational tasks. This Viewpoint paper argues for a shift from conventional chatbot paradigms toward a modular, multistep artificial intelligence (AI) agent framework that aligns closely with the pedagogical needs of medical educators. We propose a modular framework composed of specialized AI agents, each responsible for distinct instructional subtasks. Furthermore, these agents operate within clearly defined boundaries and are equipped with tools and resources to accomplish their tasks and ensure pedagogical continuity and coherence. Specialized agents enhance accuracy by using models optimally tailored to specific cognitive tasks, increasing the quality of outputs compared to single-model workflows. Using a clinical scenario design as an illustrative example, we demonstrate how task specialization, iterative feedback, and tool integration in an agent-based pipeline can mirror expert-driven educational processes. The framework maintains a human-in-the-loop structure, with educators reviewing and refining each output before progression, ensuring pedagogical integrity, flexibility, and transparency. Our proposed shift toward modular AI agents offers significant promise for enhancing educational workflows by delegating routine tasks to specialized systems. We encourage educators to explore how these emerging AI ecosystems could transform medical education.

## Introduction

The use of large language models (LLMs), such as ChatGPT, has surged in medical education, largely due to their capacity to generate answers, summaries, or clinical scenarios on demand [1]. While these tools offer convenience, most implementations remain confined to single-turn interactions with minimal contextual awareness [2]. As such, they are ill-suited to the inherently complex, multistep processes that define both clinical reasoning and medical education instructional design.

Medical education is neither linear nor static. Learners must navigate evolving information, engage in iterative reflection, and integrate feedback across diverse domains. Educators, likewise, must design learning experiences that are both structured and flexible, adapting in real time to learner needs and contextual constraints. In this Viewpoint article, we argue that advancing the role of artificial intelligence (AI) in this domain requires a conceptual and architectural shift—from chatbots to modular agents.

Elements of the proposed multistep modular agent framework are already in pilot use at our institution. For example, a history-taking application uses an agentic patient alongside companion agents that surface summaries and nudge questions when learners stall. Similar workflows have been trialed for scenario design and rubric authoring across both undergraduate and postgraduate medical education settings. We hope this Viewpoint will be useful for educators and support staff (eg, instructional designers) who are seeking structured, pedagogically aligned ways to integrate AI into their teaching and assessment practices.

## Why Single-Prompt AI Chatbots Fall Short in Complex Educational Workflows

LLMs have demonstrated strong performance in generating simulated patient responses, drafting multiple-choice questions with plausible distractors, and providing clear, conversational explanations to learners, making them valuable for isolated, language-driven tasks in medical education [1,3,4]. These interactions, however, remain fundamentally static and transactional. The AI responds once to a prompt, with limited contextual memory and no intrinsic understanding of how its output fits into a broader pedagogical framework. This architecture is not suited to the realities of medical education, which demand systems that can manage evolving learner inputs, sustain contextual awareness, and scaffold multiple interconnected subtasks. Rather than seeking access to models’ internal “reasoning,” we emphasize transparent, reviewable intermediate artifacts. For example, tables of objectives, draft scenarios, and rubric levels. These should be open to educator review at frequently defined checkpoints.

Medical educational workflows combine structure with adaptability. On one hand, structured elements such as predefined curricula, learning objectives, and assessment frameworks provide learners with a clear roadmap [5]. These components are essential for building knowledge systematically, maintaining consistency across training programs, and aligning educational outcomes with professional standards [6]. At the same time, medical education demands flexibility. Learners must continuously adapt their understanding in response to new information, clinical contexts, and feedback [7]. This dynamic process mirrors the realities of clinical practice, where patient presentations are often unpredictable, and decisions evolve as new data emerge [8].

For instance, designing a simulation scenario or a performance rubric involves more than a single step [9]. It requires iterative alignment with learning objectives, incorporation of cultural and contextual considerations, and ongoing refinement based on feedback. These are complex and adaptive processes rather than linear tasks. Relying on a single, comprehensive prompt to guide such development can result in superficial or inconsistent outputs [2]. It also places an unrealistic expectation on the prompt designer to capture all instructional nuances at once.

## Modular AI Agent Framework: A Pedagogically Aligned Alternative

To address the limitations of single-dimension chatbot systems in educational design, we propose a shift in consideration to a modular, multistep AI agent framework. An agent is a specialized AI process that operates with explicit goals, constraints, contextual inputs [10,11], and error-handling capacity. This contrasts with simple chained prompts, which merely sequence tasks without structured autonomy or memory (Table 1). Agents can operate in parallel as well as sequentially, whereas chained prompts are inherently linear.

**Table 1.** Comparison of key features of chatbots and multiagent frameworks [10,11].

Dimension	Chatbots (single or chained prompts)	Modular multiagent frameworks
Transparency	Outputs are all-in-one; intermediate reasoning often hidden	Intermediate artifacts (objectives, drafts, rubrics) explicit and reviewable
Model specialization	One model handles all subtasks; prompt engineering is the only lever	Each agent can call the model or tool best suited for its subtask
Error localization	Errors propagate; difficult to isolate or correct	Errors traceable to specific agents; easier to isolate and correct
Evaluation	Only final output assessable	Stepwise evaluation at each checkpoint
Cost	Lower per run but may require multiple retries	Higher per run (multiple agents) but more efficient for complex workflows
Pedagogical fit	Possible with skilled prompt design and oversight, but constrained by single system prompt	Agents can be designed to map systematically onto instructional frameworks
Instructional scope	Constrained by single system prompt (eg, 8000 characters in customGPT as of September 2025); must pack all instructions into one prompt; success depends on model’s ability to follow and prioritize prompts	Each agent only receives the instructions it needs for its task, reducing ambiguity and improving compliance
Scalability	Harder to adapt; changing prompts may affect entire workflow	Extensible; new agents can be added or replaced independently
Workflow structure	Sequential, linear	Supports both series (one task after another) and parallel (simultaneous subtasks) workflows

Rather than tasking a single agent with the entire instructional-design workflow, responsibilities are distributed across a pipeline of specialized agents, each optimized for its step [10,11]. These agents can invoke function calls or other tool integrations so that every agent not only reasons in natural language but also acts with the precise utilities needed to fulfill its mandate.

An *AI agentic framework* can model the expert-led, multistep process educators use in scenario design by integrating task specialization, interactive user feedback, and tool integration. Each specialized agent hands its output to the next, preserving logical and pedagogical continuity while an overarching orchestrator agent monitors the entire pipeline, coordinates handoffs, and can route back to the relevant specialist agent for further iteration when needed [12]. This intuitive structure supports extensibility through tool integration and scalability by allowing agents to be refined, replaced, or expanded based on evolving needs. In other words, pedagogy comes first: agents map intuitively onto how we already teach and assess. MEDCO (Medical Education Copilots), for example, optimizes learner-and-agent dialogue in simulated encounters [13]. In comparison, our educator-in-the-loop approach focuses sequentially on the lesson design objectives, story, tasks, and marking guide. Each draft is reviewed and approved before moving on. This enables educators to remain central to the process, reviewing and approving outputs at each stage to safeguard pedagogical integrity and contextual relevance.

## An Example in Scenario Design

We present an example of a modular AI agentic framework that offers a structured yet flexible approach to developing clinical communication scenarios. This design allows the system to emulate the collaborative workflow of a multidisciplinary education team including educators, instructional designers, content experts, and assessment specialists by assigning each phase of scenario construction to an agent optimized for that purpose.

This example system is composed of 7 AI agents, each operating within clearly defined instructional boundaries. Other than the orchestrating agent, 6 other agents function sequentially, handing off their outputs to the next agent in the pipeline to ensure continuity, coherence, and pedagogical alignment. Each agent is responsible for a specific task that reflects real-world instructional design practices, where complex deliverables are broken into manageable, interdependent stages.

The orchestrating agent oversees the entire workflow, ensuring that each specialized agent performs its task effectively and that the outputs are cohesive and aligned with the pedagogical goals. The orchestrating agent can return generated outputs from specific agents for further iteration if required.

The scenario overview agent initiates the workflow by generating a scenario title and a structured table of learning or assessment objectives. Educators are prompted to review and refine objectives before proceeding.

The storyline agent builds on the approved objectives by producing 3 patient storylines. Each includes narrative elements, such as patient background, emotional and physical concerns, hidden agendas, and sociocultural context, with explicit localisation to the Singaporean setting.

The candidate instructions agent crafts clear, structured learner instructions, including task descriptions and background details.

The surrogate patient script agent generates a detailed script to guide the simulated patient’s behavior. It incorporates behavioral cues, emotional tone, and plausible responses to candidate questions. The agent can retrieve example scripts via file searches and invoke web tools to integrate culturally relevant details.

The scoring rubric agent allows users to define their own rubric, including domains of competence and marking scales. Users can construct a table of performance criteria that aligns with the scenario’s learning objectives. The rubric incorporates score levels, descriptive anchors, and exemplar behaviors. Additionally, tool-calling and web search functionality enable the retrieval of validated rubrics to ensure alignment with existing assessment frameworks.

The final scenario agent compiles all approved components into a cohesive scenario document. It ensures consistency in formatting, terminology, and contextual accuracy, preparing the scenario for direct use or further adaptation by educators.

**Figure 1.**
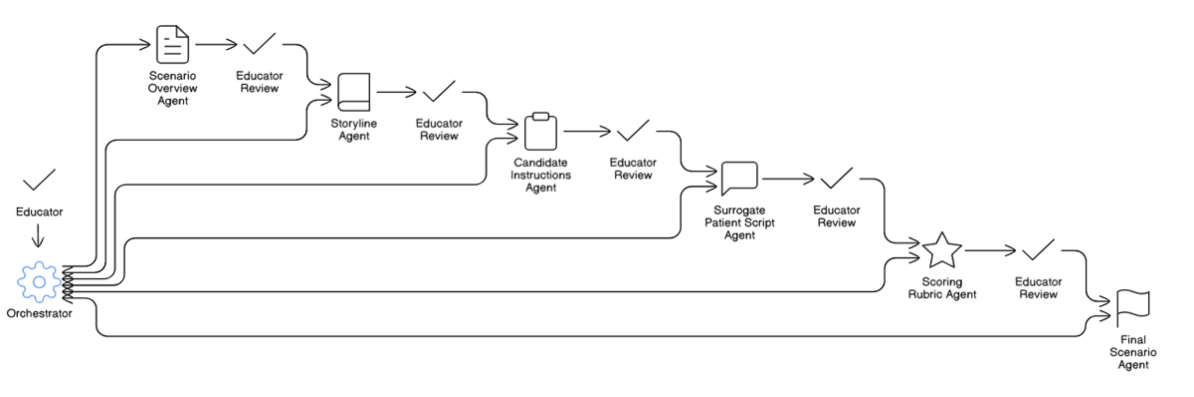
Seven-agent pipeline for scenario design with educator checkpoints. The orchestrating agent routes tasks, monitors quality, and reruns only failing steps; educators review intermediate artifacts at each gate.

Figure 1 illustrates the 7-agent pipeline with educator checkpoints and the orchestrating agent. Each handoff (represented by arrows) is schema-checked; the educator can approve or request revision before progression.

In agent-based generative workflows, the concept of a discrete “error” is less relevant than in traditional rule-based systems. Output quality is influenced by multiple factors: the specificity and clarity of user prompts, the depth of reasoning elicited by the design structure, and the availability of exemplars or templates that guide generation. Agentic frameworks allow for improved output quality, as each agent is specifically trained and instructed to perform a certain task and provided with the necessary tools to execute it effectively. Specific model types can also be selected to best fit the nature of the task, for example, reasoning, long-context, tool-use, and vision tasks. The modular framework uses these elements to produce educational artifacts that are functional, pedagogically sound, and contextually sensitive.

By breaking down the scenario design process into agent-specific tasks, the system supports a structured and transparent development process.

## Educational Theory Alignment and Pedagogical Implications

Drawing from instructional design theory, each agent’s role mirrors existing teaching and assessment practices—from objective setting to content creation to assessment alignment [14]. It also resonates with complexity theory in education, which acknowledges the nonlinear, adaptive, and emergent nature of curriculum design [8]. By assigning each AI agent a tailored reasoning strategy, such as generative models for creative storytelling or deterministic models for rubric precision, we preserve pedagogical coherence while leveraging the strengths of LLMs in context-specific ways. Moreover, the use of AI agents to build culturally localized cases (eg, context-specific language patient scripts) demonstrates how modularity can enable personalization without compromising scalability. The overall system thus acts not as a content generator, but as a structured collaborator in instructional design.

## Human-in-the-Loop: Safeguarding Pedagogical Quality and Trust

Central to our framework is the conviction that AI agents must augment—not replace—human educators. Every stage of the agentic pipeline therefore includes a mandatory checkpoint where educators review, revise, and approve an agent’s output before it is handed to the next specialist agent. Only when the reviewer is satisfied, and no further iterations are requested, does the workflow advance.

This checkpoint design yields 3 advantages. First, pedagogical assurance: continuous expert oversight guarantees that scenarios remain faithful to institutional standards, educator requirements, learner needs, and real-world clinical practice. Second, iterative flexibility: at any time, users can return to an earlier step, reopen an agent’s draft, and trigger additional refinement cycles. This bidirectional flow prevents “lock-in” to premature decisions and supports rapid improvement when curricular goals evolve. Third, transparency and trust: because every AI-generated artifact is treated as a draft, it remains open to critique rather than being accepted as a “black box” verdict. Educators retain clear visibility into the rationale behind each decision. This collaborative, human-guided process reflects traditional instructional design workflows, ensuring that final materials are shaped by pedagogical judgment rather than relying solely on algorithmic output.

To provide readers with a familiar curricular reference point, we align the proposed agentic pipeline with Kern’s [15] 6 steps of curriculum development. This alignment is not intended as a prescriptive template, but rather as an interpretive framework that illustrates how agent outputs and educator checkpoints correspond to established stages of curriculum design. In doing so, Kern’s [15] model provides conceptual coherence, while the agentic workflow demonstrates a practical means of operationalizing each step. Table 2 summarizes this mapping.

This approach reflects the collaborative nature of instructional design, where materials are developed through iterative review and decisions are guided by pedagogical judgment rather than algorithmic output alone.

**Table 2.** Agent-to-pedagogy mapping: how each agent (and the educator) contributes across Kern’s steps 1‐6.

Kern’s 6 steps	Agent and educator roles
Step 1: problem identification and general needs assessment	Scenario overview agent frames the educational problem and proposes objectives; orchestrator agent ensures consistency. Educator reviews problem framing and refines objectives.
Step 2: targeted needs assessment	Scenario overview agent structures learner-specific needs. Educator validates alignment with learner context and institutional requirements before progression.
Step 3: goals and objectives	Storyline agent translates objectives into case narratives with sociocultural context. Educator evaluates relevance, realism, and coherence of the narratives.
Step 4: educational strategies	Candidate instructions agent defines learner tasks; surrogate patient script agent operationalizes the interaction; storyline agent scaffolds narrative complexity. Educator provides iterative feedback, ensuring strategies remain pedagogically sound.
Step 5: implementation	Scoring rubric agent designs criteria for performance; final scenario agent compiles all components; orchestrator agent ensures alignment. Educator approves rubrics, validates final scenario quality, and ensures usability.
Step 6: evaluation and feedback	Scoring rubric agent embeds standards and feedback anchors; orchestrator agent supports routing to the educator for revision. Educator delivers final judgments, integrates feedback, and ensures final product is satisfactory.

## Practical Considerations

Some may argue that carefully engineered single-prompt workflows within a large language model (eg, customGPTs), can produce comparable results. Yet empirical data show that single-prompt workflows still trail agentic teams in diagnostic accuracy and bias mitigation [16-18].

Agent modularity also offers benefits that chained prompts find difficult to replicate. First, it allows the educator end user to assign each subtask to the foundation model best suited to the cognitive demands of the task. For example, a reasoning-optimized model can be used for diagnostic planning, a vision-capable model for image annotation, and a fast, low-cost model for rubric scoring.

Second, specialized agents can issue narrowly focused tool or function calls, which helps ensure stricter compliance with domain-specific instructions. An analogy illustrates this: asking a single writer to produce an entire clinical simulation, including learning objectives, patient script, scoring rubric, and feedback, in one attempt is less reliable than assigning each section to professionals who have the right expertise and references. Modularity gives an AI pipeline that same division of labor advantage.

Third, modularity localizes failure and simplifies evaluations. Breaking the workflow into a series of small, purpose-built agents lets educators spot problems exactly where they occur, verify the quality of each step in isolation, and rerun or update only the affected agent without disturbing the rest of the pipeline.

While the modular-agent approach offers clear advantages, it is not without trade-offs. Stitching together multiple agents raises implementation complexity, especially in low-resource or nontechnical settings. Here, “vibe coding,” a visual and block-based approach to wiring agents, tool calls, and routing rules, is helping to lower the barrier for educators who do not have coding experience [19]. Vibe coding allows educators to create educational apps by instructing AI in natural language. Any educator who hopes to pivot toward AI-augmented end-to-end educational workflows must begin with careful process decomposition. The instructional journey should be mapped into discrete steps, with the cognitive and technical requirements of each step clearly specified [20,21]. There are several practical implementation choices to consider. The input and output schemas between agents are standardized at each step with simple templates to ensure minimum quality standards. Educators are provided with “prompt cards” documenting instructions, data sources, tools, and approvals, with any changes logged for accountability. All drafts are automatically logged with source, model, prompt card, and a summary that enables traceability. Clear acceptance criteria guide fallback or rollback when a step underperforms, allowing repair or educator-initiated rewrites without restarting the pipeline. Versioning and update policies are also in place. Runs typically complete within minutes, with token use and costs managed by combining efficient models for routine steps with reasoning models for critical alignment.

## Limitations

This Viewpoint advances a conceptual and architectural position supported by early pilot deployments; however, we have not yet conducted prospective controlled studies to compare modular multiagent pipelines with single-step chatbots in terms of their impact on learner outcomes.

## Conclusion: Rethinking AI’s Role in Medical Education Together

Moving beyond chatbot paradigms opens new possibilities for the use of AI in medical education. Modular, agent-based systems can mirror the sophistication of human instructional design, support iterative feedback loops, and preserve important contextual nuance. Their true value lies not in replacing educator labor, but in amplifying educator intent. Realizing this potential requires investment in systems that prioritize transparency, alignment, and human AI collaboration.

One promising step is the shift toward purpose-built agent ecosystems. Rather than relying on single-prompt chatbots, educators can begin by decomposing the instructional process, mapping each step, and identifying where a specialized agent, equipped with the appropriate model, data access, and tool-calling privileges, might help relieve cognitive load or automate repetitive tasks. Delegating routine activities to autonomous agents allows human experts to reclaim valuable time for higher-order mentoring, empathy, and creative pedagogy. Embracing this agentic mindset can help AI feel less like a black box chatbot and more like a transparent, collaborative partner that transforms tedious processes into opportunities for deeper learning and innovation.

We invite educators who are curious and committed to innovation to join us in exploring and shaping these agentic ecosystems together.

## Abbreviations

AI: artificial intelligence
LLM: large language model
MEDCO: Medical Education Copilots
